# Novel High-throughput Approach for Purification of Infectious Virions

**DOI:** 10.1038/srep36826

**Published:** 2016-11-09

**Authors:** Kevin T. James, Brad Cooney, Kate Agopsowicz, Mary Ann Trevors, Adil Mohamed, Don Stoltz, Mary Hitt, Maya Shmulevitz

**Affiliations:** 1 Department of Medical Microbiology and Immunology, Li Ka Shing Institute of Virology, University of Alberta, Edmonton, Alberta, Canada; 2 GE Healthcare, LifeSciences, Marlborough, MA, US; 3 Department of Oncology, University of Alberta, Edmonton, Alberta, Canada; 4 Department of Microbiology and Immunology, Dalhousie University, Halifax, Nova Scotia, Canada

## Abstract

Viruses are extensively studied as pathogens and exploited as molecular tools and therapeutic agents. Existing methods to purify viruses such as gradient ultracentrifugation or chromatography have limitations, for example demand for technical expertise or specialized equipment, high time consumption, and restricted capacity. Our laboratory explores mutations in oncolytic reovirus that could improve oncolytic activity, and makes routine use of numerous virus variants, genome reassortants, and reverse engineered mutants. Our research pace was limited by the lack of high-throughput virus purification methods that efficiently remove confounding cellular contaminants such as cytokines and proteases. To overcome this shortcoming, we evaluated a commercially available resin (Capto Core 700) that captures molecules smaller than 700 kDa. Capto. Core 700 chromatography produced virion purity and infectivity indistinguishable from CsCl density gradient ultracentrifugation as determined by electron microscopy, gel electrophoresis analysis and plaque titration. Capto Core 700 resin was then effectively adapted to a rapid in-slurry pull-out approach for high-throughput purification of reovirus and adenovirus. The in-slurry purification approach offered substantially increased virus purity over crude cell lysates, media, or high-spin preparations and would be especially useful for high-throughput virus screening applications where density gradient ultracentrifugation is not feasible.

Most laboratories use one of three methods to prepare virus for experimentation. The most rapid approach involves using crude cell lysates or cell culture media from virus-infected cells. To generate lysates, cells are commonly lysed by freeze-thaw, debris is removed by low speed centrifugation, and supernatants are then used for experimentation. While this approach is quick and permits simultaneous high-throughput comparisons of a large sundry of viruses, it suffers from high contamination with non-virus factors that may affect experimental outcomes (Table 1). For example, cellular proteases could degrade virus, while cytokines could alter cell signalling and affect susceptibility to viruses. An intermediate-purity virus preparation is achieved by adding a high speed ultracentrifugation step. Viruses are either pelleted directly, pelleted through a low-density sucrose solution to reduce aggregation, or floated above a high-density sucrose cushion. This straightforward 60-minute step can eliminate many soluble host factors, but suffers from continued contamination with non-viral proteins. To achieve ultimate virus purity, the most commonly used method in a laboratory setting is density gradient ultracentrifugation. Most typically, gradients of CsCl, sucrose, or iodixanol are used to separate viruses from host contaminants. This approach also allows for the separation of complete- from incompletely assembled or genome-devoid (empty) virions. The pitfalls of equilibrium density ultracentrifugation are that it is time consuming (typically 2–3 days) and restricted in sample number (6 samples per ultracentrifuge). The choice among these three methods in laboratories is therefore dictated by trade-offs between time savings and productivity versus need for purity.

Our laboratory sought a straightforward and affordable method to purify many viruses at once. Our research explores molecular aspects of reovirus replication, and makes routine use of spontaneous or reverse-engineered mutants to manipulate virus phenotypes. A limitation to high-throughput screening of virus mutants is the absence of a productive virus purification strategy that also provides high purity. Since reovirus is sensitive to antiviral and stress signalling, and can loose infectivity by premature proteolysis, the purification method needs to remove cellular contaminants that could affect virus infectivity and introduce confounding effects. Moreover, a high-throughput approach needs to handle more samples than traditional density gradient ultracentrifugation can support.

The growing interest in using viruses for gene therapy, vaccines, or oncolytic cancer treatments has required development of new chromatography-based virus purification methods that are consistent, scalable and GMP manufacturing grade. Reovirus is among several viruses currently being explored in cancer immunotherapies, so industrial-grade methods for purifying and quantifying reovirus on a large scale are being developed for the purpose of human clinical testing^1^,^2^. For example, highly purified reovirus was achieved using a combination of sequential filtration, diafiltration, ion-exchange chromatography, and gel permeation chromatography. Chromatography methods have also been developed for commonly-used viruses such as adenovirus, adeno-associated virus, or lentivirus, and are well-summarized in a review by Segura *et al*.^3^. For example, distinct ionic exchange resins can selectively enrich for viruses based on unique net surface charge. Alternatively, affinity chromatography methods exploit the natural ability of some viruses to bind cell surface molecules such as heparin or sialic acids, or make use of antibodies to enrich specific viruses. Finally, given the large size of viruses relative to most cellular factors, size exclusion chromatography is commonly applied as a sole- or complementary step during industrial virus purification. Chromatographic methods therefore offer a growing alternative to gradient ultracentrifugation for high purity virus preparation, especially compatible with the industrial setting. Chromatographic methods may, however, may be less-compatible with funding, equipment, and expertise available in standard virology laboratories. Furthermore, these methods are not well-suited for simultaneous purification of many virus variants, mutants, or isolates.

Our quest to develop a high-throughput method for purifying reovirus was achieved using Capto Core 700 chromatography resin (GE Healthcare). The octylamine ligands within Capto Core 700 ‘beads’ are designed to have both hydrophobic and positively charged properties that can trap molecules under 700 kilodaltons. Since viruses exceed 700 kDa, and since the bead exterior is inactive, Capto Core 700 permits purification of viruses by size exclusion. Application notes from the manufacturer suggest that Capto Core 700 chromatography is effective at purifying influenza A virus from infected mammalian cell cultures^4^, and human papilloma virus-like particles from insect cells (GE Healthcare application notes 29-1037-62 AA, 29-0003-34 AA, and 29-0983-01 AB). Moreover, Zhao *et al*.^5^ recently demonstrated that enterovirus71 can be effectively purified using Capto Core 700 chromatography^5^. These studies suggested that Capto Core 700 resin was compatible with purification of diverse virus families including *Picornaviridae* (non-enveloped, +ssRNA genome), *Orthomyxoviridae* (enveloped, -ssRNA segmented genome), and *Papillomaviridae* (non-enveloped, dsDNA genome).

Our current analysis demonstrates that Capto 700 chromatography is also compatible with reoviruses, which contain segmented dsRNA genomes, and adenovirus, which contain dsDNA genomes. Moreover, we demonstrate that the Capto 700 resin can be transformed into a “slurry approach”, in which crude virus-infected lysates are rotated with resin end-over-end for one hour to remove cellular contaminants. The slurry approach provides a rapid method to purify reovirus without need of chromatography equipment, and can easily be used to purify many viruses simultaneously. Capto 700 slurry purification offers a practical approach for virology laboratories to achieve increased virus purity over crude cell lysates, media, or high-spin preparations. This method is especially useful for high-throughput virus screening applications, where density gradient ultracentrifugation approach is not feasible.

## Results

### Reovirus is effectively purified by Capto Core 700 chromatography

**Figure 1:**
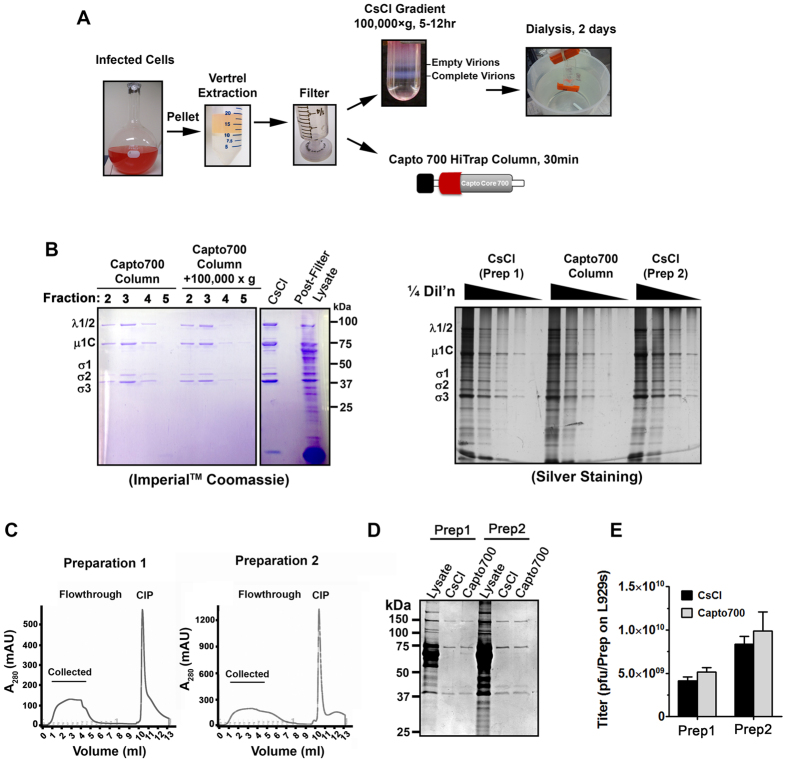
Reovirus is effectively purified by Capto Core 700 chromatography. **(A)** Diagrammatical depiction of CsCl gradient versus Capto Core 700 chromatography-based reovirus purification strategies. **(B**, left) Equal volumes of unpurified reovirus-infected lysate (Poster-Filter Lysate), CsCl purified reovirus (CsCl), or fractions 2–5 obtained following Capto Core 700 chromatography were subjected to SDS-PAGE and Coomassie staining. Location of reovirus structural proteins are indicated. **(B**, right) Two independent preparations of CsCl purified reovirus (CsCl) and and independent preparation of Capto700 column purified virus were assessed by SDS-PAGE and silver staining. Equivalent OD260 were loaded as starting concentration for each virus, followed by 1/4^th^ dilutions. **(C–E)** Two independent large-scale reovirus preparations were subjected to CsCl gradient ultracentrifugation versus Capto Core 700 chromatography. **(C)** Chromatograms demonstrate milli- absorption units (mAU) at 280 nm for each 1 ml fraction during collection and cleaning-in-place (CIP). **(D)** SDS-PAGE and Coomassie staining show purify and recovery of reovirus preparations. **(E)** Plaque titration shows infectivity of reovirus preparations.

To determine if the Capto Core 700 contaminant-capture chromatography method could be used to purify reovirus, we divided a large spinner culture of reovirus-infected L929 cells into two portions and subjected them either to traditional CsCl gradient ultracentrifugation or Capto Core 700 column chromatography (Fig. 1A). Cells were exposed to reovirus at a multiplicity of Infection equal to 3, and collected when approximately 60–70% of cells stained positive with trypan blue. Under these conditions, a majority of reovirus remains cell-associated and so can be concentrated by low-speed centrifugation. The cell pellet was resuspended in virus stabilization buffer (150 mM NaCl, 15 mM MgCl_2_, 10 mM Tris pH 7.4). For non-enveloped viruses such as reovirus, organic solvent extraction liberates virus particles from cell debris and removes lipids without hindering virion stability. Reovirus-infected cell pellets were therefore once-extracted with Vertrel^®^ XF, an environmentally friendly organic solvent previously optimized by Mendez *et al*.^6^ for reovirus extraction^6^. The aqueous phase was passed through a 0.45 μm regenerated cellulose GD/X filter and divided into two portions. One portion was layered on a 1.2–1.4 g/cc gradient of CsCl, subjected to high speed ultracentrifugation, and dialyzed against virus stabilization buffer. The second portion was passed through a Capto Core 700 column using the AKTA Start liquid chromatography system.

As expected, CsCl density gradient ultracentrifugation produced highly pure reovirus particles as indicated by SDS-PAGE and Imperial^TM^ Coomassie blue staining (Fig. 1B, left) or silver staining (Fig. 1B, right). Dominant structural proteins of reovirus including outer capsid σ3 and μ1C proteins, and inner capsid σ2 and λ1 proteins, were clearly separated from remaining host proteins visible in the post-filter (but pre-purification) lysates. Density gradient ultracentrifugation also produced a concentrated sample of approximately 1 millilitre (from 5–7 ml input lysate), and permitted separation of complete virions from genome-devoid (empty) virus particles (Fig. 1A).

During Capto Core 700 column chromatography, reovirus proteins predominated in the first 2–5 1-millilitre elution fractions (Fig. 1B, left). Purity of reovirus proteins seemed comparable between Capto Core 700 chromatography versus CsCl gradient ultracentrifugation methods (Fig. 1B, right). Reovirus proteins were less concentrated following chromatography due to dispersion among several fractions. When fractions were further concentrated by high-speed centrifugation for 1 hour, there was limited loss of virus protein intensity. High speed centrifugation therefore offers a way to concentrate reovirus if needed, but importantly suggests that reovirus bands represent whole virions rather than soluble virus proteins.

Capto Core 700 chromatography was then repeated with two additional independent medium-scale (500 ml L929 spinner culture) preparations to assess reproducibility and virus integrity. Chromatograms of absorbance at 280 nm consistently showed peaks at flowthrough fractions 1–5, which were therefore collected and pooled (Fig. 1C). Each preparation was also subjected to matched CsCl gradient ultracentrifugation, but this time final volumes were equalized in order to compare total reovirus capture efficiency. For both preparations, reovirus protein purity and total capture efficiency were similar between the two purification strategies (Fig. 1D). Moreover, both CsCl gradient ultracentrifugation and Capto Core 700 chromatography produced equivalent reovirus titers (within a given preparation), as assessed by standard plaque titration on L929 cells (Fig. 1E). Taken together, these findings suggest that size exclusion chromatography is an effective method to purify reovirus and could provide a time-saving approach when laboratories are not concerned about separating complete- from empty- virus particles.

### Capto Core 700 resin can be applied in-slurry to purify reovirus

With standard gel filtration (size exclusion chromatography), molecules of smaller size spend more time penetrating pores of the stationary phase, and therefore exhibit higher retention (slower elusion) relative to larger molecules. In contrast, the ligand-activated pores of Capto Core 700 are touted as having electrostatic and hydrophobic interactions that “capture” molecules under 700 kDa. We reasoned that if contaminants were truly “trapped” within Capto Core 700 resin, then it might be possible to purify reovirus from infected cell-lysates in suspension. A suspension purification method would become independent of chromatography equipment and therefore be compatible with high-throughput virus purification.

**Figure 2:**
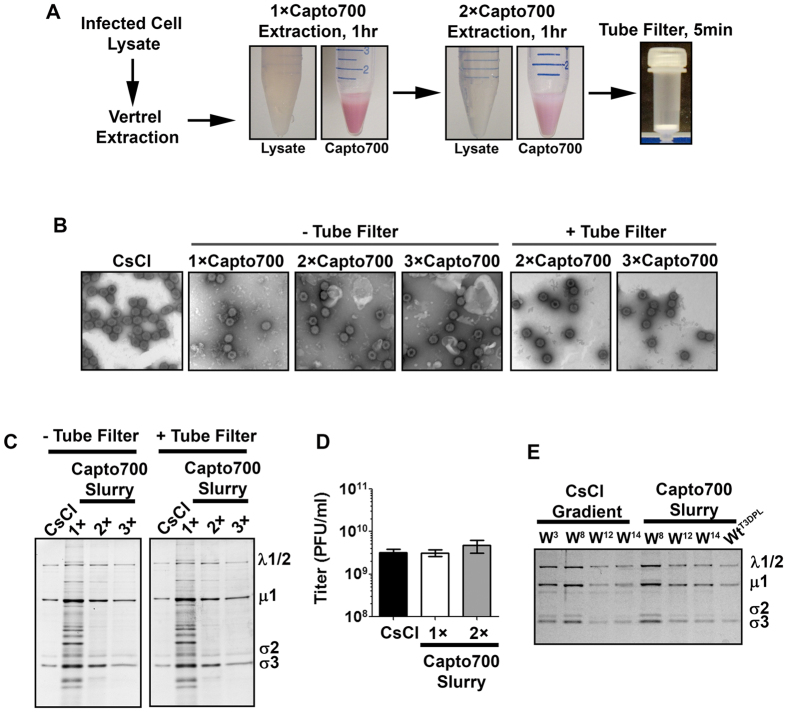
Capto Core 700 resin can be applied in-slurry to purify reovirus. **(A)** Photographs depict steps of Capto Core 700 in-slurry reovirus purification as described in text. **(B–D)** Reovirus particles were purified by CsCl gradient ultracentrifugation or by 1–3 rounds of in-slurry purification with Capto Core 700 resin in the absence or presence of microcentrifuge filtration to remove residual resin. Reovirus particles purity, recovery, and infectivity was assessed by **(B)** electron microscopy, **(C)** SDS-PAGE and Coomassie staining, and **(D)** plaque titration. **(E)** Several wild isolates of reovirus obtained from primary effluent by Dr. Pang at the Alberta Provincial Laboratories (W^3^, W^8^, W^12^, and W^14^) or our laboratory strain of wild-type reovirus dearing (WT^T3DPL^) were propagated in L929 cells and then purified by CsCl gradient ultracentrifugation or two rounds of Capto Core 700 in-slurry methods and compared by SDS-PAGE and Coomassie staining.

The Capto Core 700 slurry virus purification approach was attempted on medium-scale (500 ml) L929 spinner culture lysates once-extracted with Vertrel^®^ XF. Again, one-half of the post-extracted lysates were subjected to traditional CsCl gradient ultracentrifugation as basis for comparison to new methods. The second half of the post-extracted lysates was mixed with a 50% slurry of Capto Core 700 resin in virus stabilization buffer, rotated end-end for 1 hour at room temperature, then separated from resin by centrifugation (Fig. 2A). Up to three rounds of extraction with Capto Core 700 resin were evaluated. We used sequential extractions, rather than simply increasing the resin volume, to promote successive distribution between resin versus soluble fractions. Photographs of lysates and resin post-separation show that the resin was capable of removing contaminants such as phenol red pH indicator introduced from cell culture media. Furthermore, the lysate progressively clarified to almost-clear by the second sequential extraction.

The integrity and purity of reovirus purified by the Capto Core 700 slurry approach was examined by negative stain transmission electron microscopy (EM). It was evident that after 1, 2, or 3 rounds of slurry purification, reovirus particles remained intact similar to CsCl gradient ultracentrifugation (Fig. 2B). However, with successive addition of Capto Core 700 resin, there appeared to be an increase of large debris perhaps attributed to the resin itself. Virions were effectively clarified through simple MicroSpin columns with a 10 micron frit for bed support (Fig. 2B, +Tube Filter). The residual small particulates in virus preparations following MicroSpin column filtration may represent remnants of the resin rather than proteinaceous contaminants since SDS-PAGE gel electrophoresis showed that 2–3 rounds of Capto Core 700 slurry purification produced similar virus purity and capture efficiency relative to CsCl gradient purification (Fig. 2C). Furthermore, MicroSpin column filtration did not lower virus yields. Importantly, virus titers were equivalent between CsCl gradient and Capto Core 700 slurry approaches (Fig. 2D). The quality of several independent preparations of natural isolates of reovirus (Wild, W) and our laboratory isolate of wild-type reovirus serotype 3 Dearing (Wt) seems comparable between CsCl gradients versus Capto Core 700 Slurry approaches (Fig. 2E). Altogether, results suggest that optimal large-scale slurry-based purification of reovirus should include 2–3 clarifications with Capto Core 700 slurry, followed by an optional pass through polyethylene filter columns to remove large particulates.

### High-throughput reovirus purification using Capto Core 700 in-slurry approach

**Figure 3:**
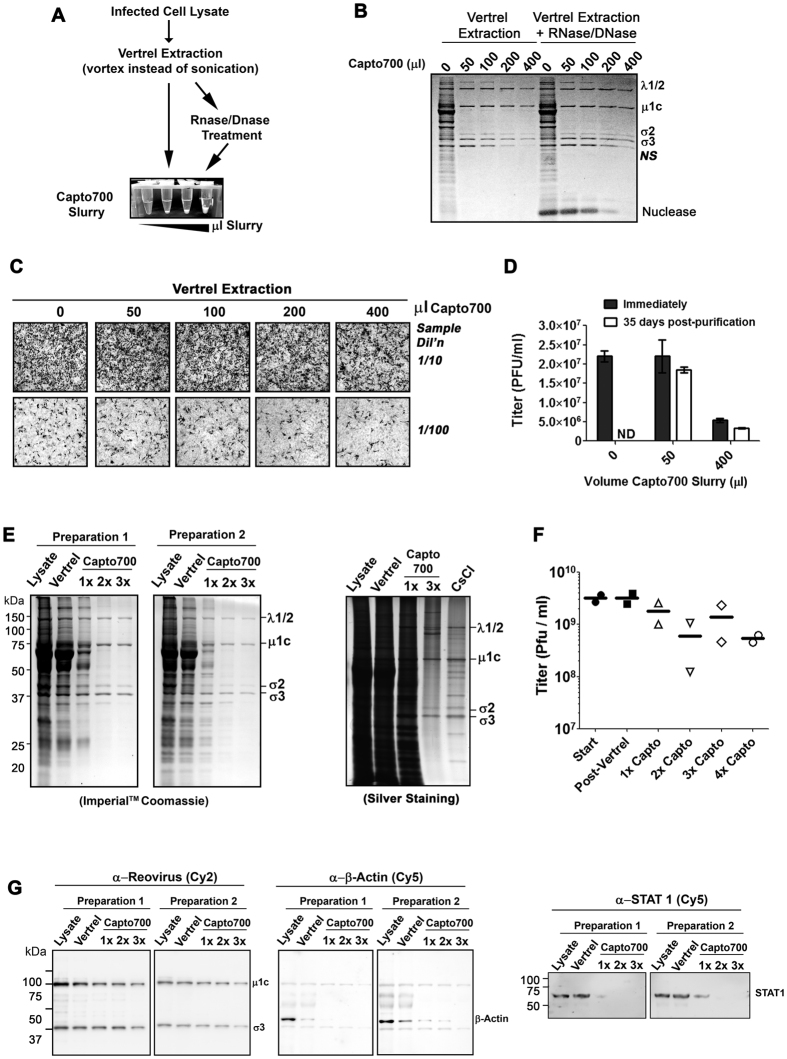
High-throughput reovirus purification using Capto Core 700 in-slurry approach. **(A–D)** 10 cm^2^ wells of confluent reovirus-infected L929 cells were once-extracted with Vertrel XF, incubated with or without RNase/DNase then subjected to one round of in-slurry purification with 0, 50, 100, 200 or 400 μl of Capto Core 700 resin (50% slurry in virus dilution buffer) for 1 hour at room temperature. **(B)** Reovirus virion recovery and purity were assessed by SDS-PAGE and Coomassie blue staining. **(C)** L929 cells were exposed to purified reovirus samples from (**B**) at indicated dilutions. The infectivity of samples was then monitored at 18 hours post-infection by immunocytochemical staining with polyclonal anti-reovirus antibodies. **(D)** Plaque forming titers for samples in (**B**) were determined immediately post-purification or following 35 days of storage at 4 °C. **(E–G)** Four million reovirus-infected Ras-transformed NIH3T3 cells were once-extracted with Vertrel XF then subjected to 0–4 rounds of 45-minute end-over-end incubation with 50 μl of 50% Capto Core 700 resin slurry at room temperature. Results from two independent preparations are provided. **(E)** SDS-PAGE and Coomassie blue staining (left) or silver staining (right) of unpurified infected lysates (lysate), Vertrel-extracted samples (Vertrel), or samples exposed to 0–4 rounds Capto Core 700 resin (Capto700). **(F)** Infectivity was assessed by plaque titration. **(G)** Samples from (**E**) were subjected to western blot analysis. The same membrane was first immunoblotted with polyclonal rabbit anti-reovirus antibodies and Cy2-conjugated anti-rabbit secondary antibodies, then re-probed with mouse anti-β-actin antibodies and Cy5-conjugated anti-mouse secondary antibodies. A separate membrane was blotted with rabbit anti-STAT1 and Cy5-conjugated anti-rabbit secondary antibodies. Protein bands were visualized at respective wavelengths using an ImageQuant LAS 4010 imager (GE Healthcare).

Having successfully purified reovirus in suspension using the Capto Core 700 resin in a medium scale preparation, we next optimized the application of this approach for small-scale but high-throughput applications (Fig. 3A). Reovirus-infected L929 cell lysates from 1 × 10^6^ cells were subjected to extraction with Vertrel^®^ XF. To reduce processing time and complexity, samples were not sonicated but rather extracted by addition of Vertrel, vortexing, and centrifugation. The top aqueous phase was then mixed end-over-end with various amounts of Capto Core 700 resin (0–400 μl) in suspension for 1 hour at room temperature. The resin was removed by brief centrifugation, and samples were subjected to protein electrophoresis and Imperial^TM^ Coomassie blue staining (Fig. 3B). Vertrel extraction followed by in-slurry purification with Capto Core 700 resin was effective at maintaining high yields of reovirus proteins with minimal contamination by cellular proteins. We did note, however, that a non-specific (NS) protein persisted in preparations unless large volumes of resin were used. Additional treatment with RNase and DNase prior to in-slurry purification did not visibly improve virus purity.

The integrity of reovirus preparations was then tested by two methods. First, L929 cells were exposed to unpurified lysates or lysates cleared by Capto Core 700 resin, then stained immunocytochemically (ICC) at 15 hours post-infection for reovirus proteins expression (Fig. 3C). ICC staining suggested that lysates extracted with 50 μl Capto Core 700 resin infected a similar number of cells relative to unprocessed lysates, but that progressively higher volumes of Capto Core 700 resin resulted in reducing virion infectivity. Some samples were also subjected to plaque titration either immediately following purification, or following 35 days of storage at 4 °C (Fig. 3D). Results showed minimal loss of infectivity when reovirus was purified with 50 μl of Capto Core 700 resin (Fig. 3D). Importantly, purified reovirus retained infectivity over prolonged storage at 4 °C, indicating limited proteolysis commonly associated with crude purification procedures.

The in-slurry Capto Core 700 purification method was then further validated and optimized. To test applicability to alternative cell lines, we used reovirus-infected NIH3T3 fibroblasts transformed with constitutively active Ras oncogene. Ras-transformed NIH3T3 cells are highly susceptible to reovirus^7^,^8^,^9^ and can reach high cell density, supporting efficient reovirus production. Starting with four times more cells then L929 cells (i.e. 4 × 10^6^ cells/preparation), we evaluated whether 1–3 sequential in-slurry purifications, each for 45 minutes rotating end-over-end, could further promote virus purity. Virus purity was monitored by Imperial^TM^ coomassie- (Fig. 3E, left) or silver staining (Fig. 3E, right). Relative to unpurified lysates or Vertrel extraction alone, 2–3 sequential in-slurry purifications successfully removed most cellular contaminants including the aforementioned non-specific (NS) protein. Silver staining showed some contaminating bands and smears in virus preparations purified by both CsCl gradient ultracentrifugation and Capto core 700 in-slurry approaches. Plaque titration demonstrated retained infectivity of purified reovirus (Fig. 3F). For optimal time-savings versus purity and infectivity, two rounds of in-slurry purification seemed optimal.

Western blot analysis was used to assess levels of specific viral and cellular proteins at higher sensitivity. Since only a portion of reovirus proteins within infected cells are assembled into whole virions, we were not surprised that intensities of reovirus major structural proteins μ1c and σ3 were reduced following in-slurry purification (Fig. 3G). Cytoskeleton-associated β-actin was effectively removed by Vertrel extraction, and then further cleared by in-slurry purification. Importantly, immunoblot analysis suggests that the Capto Core 700 in-slurry step was critical for removing the cytoplasmic signaling molecule STAT1 (signal transducer and activator of transcription 1), a representative cytosolic and moderately-sized (87 KDa) cellular protein.

### Purification of adenovirus in high-throughput using Capto Core 700 in-slurry approach

**Figure 4:**
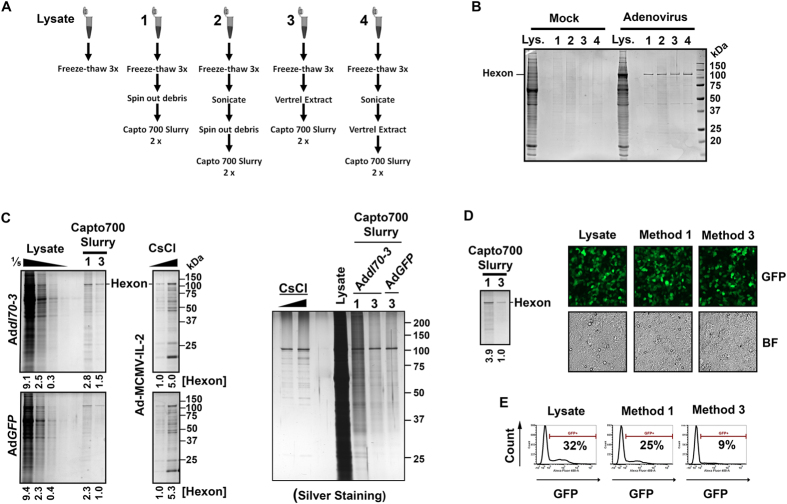
Purification of adenovirus in high-throughput using Capto Core 700 in-slurry approach. (**A,B**) 10 cm2 equivalence of HEK293 cells were left uninfected (mock) or infected with adenovirus Addl309 (containing E1 and E3 regions of Ad5) until cytopathic effect exceeded 90%. Mock- and adenovirus infected lysates were frozen and thawed three times, then either left unpurified (lysate) or subjected to 4 alternative purification strategies as summarized in (**A**). (**B**) Recovery and purity of adenovirus was assessed by SDS-PAGE and Coomassie staining. Locations of major adenovirus proteins are indicated. (**C**) HEK293 cells infected with Addl70-3 (no transgene) or AdGFP (GFP transgene) and left unpurified (lysate) or purified by methods 1 or 3 as depicted in (**A**). SDS-PAGE and Coomassie staining (left) or silver staining (right) shows recovery and purity of samples purified by Capto Core 700 resin compared to Ad-MCMV-IL-2 (contains an IL-2 transgene) CsCl purified adenovirus. Serial dilutions of 1 in 5 were provided for lysates and Ad-MCMV-IL-2. Densitometric analysis for quantities of hexon proteins is provided. (**D**) Coomassie blue staining shows relative adenovirus hexon proteins in purified samples. HEK293 cells were exposed to equivalent dilutions of AdGFP lysates or samples purified by methods 1 or 3 and at 24 hours post infection, visualized by fluorescence microscopy (GFP) and bright field (BF). (**E**) Similar to (**D**) but all samples were diluted further by one-fifth and virus-infected cells were quantified flow cytometry. Percent of cells positive for GFP expression are indicated.

To determine if the Capto Core 700 in-slurry method could extend to other viruses, we evaluated the method for purification of adenovirus. Adenovirus is a common cause of respiratory illnesses though generally causing only mild symptoms. Adenoviruses are frequently used as vectors for gene expression and are under investigation as oncolytic virotherapies. A high-throughput purification strategy for adenoviruses would facilitate rapid comparison of natural and engineered adenovirus variants. Like reoviruses, adenoviruses are non-enveloped and medium size particles (80–100 nm). In contrast to reoviruses, adenoviruses have a DNA genome and replicate in the cell nucleus. To determine if the Capto core 700 in-slurry approach was applicable to purification of adenovirus, we subjected mock- or adenovirus-infected HEK293 human embryonic kidney cells to various conditions such as freeze-thaw, sonication, and/or Vertrel extraction, followed by two rounds of Capto Core 700 slurry clarifications (Fig. 4A). Coomassie blue staining showed effective removal of cellular proteins from mock- and infected- cell lysates especially in the absence of sonication. Similar persistence of dominant adenovirus structural proteins such as hexon and protein V was found between conditions 1–5 (Fig. 4B). Note that as with reovirus, only a portion of viral proteins produced in infected cells are expected to be assembled into whole virions and therefore recovered from total cell lysates.

The best two conditions (freeze-thaw with or without Vertrel extraction prior to Capto Core 700 in-slurry purification) were then applied to two additional first generation (E1 and E3- deleted) adenovirus constructs (Fig. 4C). Addl70–3 contains no transgene insert, while AdGFP encodes green fluorescent protein. Purified viruses were compared to serial dilutions (1/5) of unpurified lysates by Coomassie blue staining. Serial dilutions (1/5) of another adenovirus construct (AdML-4 which expresses IL-2) previously purified by CsCl gradient ultracentrifugation was also included to show the electrophoretic banding pattern of adenovirus major and minor structural proteins. Virion purity was assessed by Imperial^TM^ coomassie- or silver staining. Results suggest that inclusion of Vertrex extraction provides added virion purity, as indicated by further removal of proteins absent from CsCl purified virions. Vertrel extraction did however slightly reduce recovery, as indicated by a 2–3 fold reduction in hexon protein band intensity. Silver staining suggests that adenovirus purified by Capto Core 700 in-slurry is less pure than adenovirions purified by CsCl density ultracentrifugation, and therefore the in-slurry approach is most valuable for adenovirus screening applications where ultracentrifugation or commercial adenovirus purification columns are not feasible.

Plaque titration was performed to determine infectious titers of adenovirus lysates and purified samples after storage at 4 °C or −80 °C (Table 2). Adenovirus titers were similar pre- versus post-purification, indicating reproducible recovery of infectious adenovirus virions regardless of the specific construct used for analysis. The GFP-expressing adenovirus construct (AdGFP) was also used as a complementary approach to evaluate the infectivity of recovered virions. Purified virions or unpurified lysates were applied to fresh monolayers of HEK293 cells, and the extent of infection was monitored at 24 hours post-infection by fluorescence microscopy (Fig. 4D) and flow cytometry (Fig. 4E). Method 1 (freeze-thaw plus Capto Core 700) produced equal infectivity to lysates alone, while method 3 (inclusion of Vertrel extraction) resulted in approximately 4-fold lower levels of hexon and infectious virions. These results demonstrate that infectivity-per-particle is preserved following extraction with Vertrel and purification with Capto Core 700 slurry; albeit Vertrel extraction seems to reduce the number of particles by 2–4 times (Fig. 4C–E). Given the time savings of the in-slurry approach, even a 20% recovery rate would produce ample virions for experimentation and is most likely adequate. Furthermore, method 1-purified adenovirus had 5-fold lower hexon levels than unpurified samples (Fig. 4C) but similar infectivity (Fig. 4D,E), suggesting that a majority (~80%) of hexon polypeptides were not assembled into virions within infected cell lysates, and that Capto Core 700 resin effectively removed unassembled virion proteins.

## Discussion

Here we present a new method for purifying either reovirus or adenovirus samples in high-throughput format. The in-slurry Capto Core 700 purification method requires less than 3 hours, can be simultaneously applied to many samples, is technically straightforward, and produces virion purity and infectivity indistinguishable from density gradient ultracentrifugation as determined by both EM and gel electrophoresis analysis. The capto Core 700 resin can be removed by centrifugation, or completely clarified through MicroSpin columns. The in-slurry method further eliminates the need for chromatography equipment, reduces contamination between samples, and is easily scaled for small, medium, or large preparations. It should be noted that for cost saving purposes, the resin is advertised as being reusable. Although we did successfully reuse the Capto Core 700 chromatography columns 2–3 times initially (data not shown), we opted for single-use applications to avoid cross-contamination between virus mutants. The main foreseeable drawback to purification through Capto Core 700 columns or in-slurry, is that genome-devoid (empty) virions are not eliminated. Conversely, since mutations might affect the efficiency of genome packaging, the inclusion of empty virions might help uncover previously overlooked phenotypes.

Our study focused on reoviruses and adenoviruses. GE Healthcare application notes suggest that Capto Core 700 chromatography can also be applied to influenza A virus and human papilloma virus like particles (GE Healthcare application notes 29-1037-62 AA, 29-0003-34 AA, and 29-0983-01 AB). The in-slurry adaptation for high-throughput purification is therefore likely to work for these and other viruses as well. Once optimized for individual virus families, Capto Core 700 chromatography or in-slurry methods would enable large numbers of virus samples to be rapidly compared for phenotypes such as infectivity. For example, the in-slurry method could easily be applied to 24–30 samples in a standard centrifuge or even multiples of 96 samples in 96-well plates. We envision this approach being applied similar to routine high-throughput purification of plasmids and RNA. The 0.45 μm filtration step avoids microbial contamination during large- and medium- scale purifications, but high-throughput in-slurry purification using Capto Core 700 resin will likely benefit from precautions such as aliquoting and freezing of virus preparations or use of sterile reagents. Finally, an increasing number of laboratories use viruses as molecular tools, for example to deliver genes or interfering RNAs using adenovirus, adeno-associated virus, or lentivirus vectors. The rapid in-slurry virus purification strategy may accelerate these routine procedures.

The development of the Capto Core 700 chromatography and in-slurry methodology is expected to contribute significantly to the efforts of those having interest in reovirus or adenovirus biology. For example, many reovirus protein functions have been characterized by comparing phenotypes among natural reovirus variants, reovirus genome reassortants, and laboratory derived reovirus mutants^10^,^11^,^12^,^13^,^14^,^15^,^16^,^17^,^18^,^19^. In conjunction with reverse genetics approaches for these viruses^20^,^21^,^22^,^23^,^24^,^25^, it is now possible to design copious virus mutants, purify them in-slurry, and make rapid comparisons of virus proteins, virion structure, and virus replication. Furthermore, both reovirus and adenovirus are heavily pursued as oncolytic therapies. We and others have demonstrated that reovirus mutants exhibit differences in their oncolytic activities, and that second-generation reovirus variants may possess improved oncolytic potency^17^,^19^,^26^,^27^. We have found the Capto Core 700 slurry method to be extremely useful for purifying and comparing various natural isolates (Fig. 2E) and laboratory variants (data not shown) for oncolytic activity. For transgene-containing adenovirus vectors, this method of purification should yield vector preparations free of transgene product which can confound analysis of vector activity, in little more time than required to prepare a crude lysate. Furthermore, this strategy should facilitate screening populations of vectors re-targeted by “shotgun-style” insertion of sequences into genes encoding fiber or other capsid proteins. Importantly, this approach should provide a quick method to prepare immune-modulating viruses that are free of cellular proteins such as cytokines, calreticulin and HMGB1 that are known to alter immunologic responses.

## Materials and Methods

### Virus purification by CsCl, Reovirus purification on Capto Core 700 chromatography columns, and Capto Core 700 slurry

Protocols for virus purification by CsCl, by Capto Core 700 chromatography columns, and by Capto Core 700 in-slurry approach are described in the Supplementary Materials and Methods.

### Virus Stocks

Reovirus serotype 3 Dearing laboratory strain was generously provided by Patrick Lee (Dalhousie University, Canada). Natural (wild) isolates of reovirus were obtained from Edmonton primary effluent collected and identified by Xiao-Li Pang (Alberta Provincial Laboratory, Canada). Adl70-3 was generated by previously described methods (27). AdGFP was generated by recombining an dE1-deleted, E3-deleted adenovirus vector backbone with an expression plasmid containing the murine CMV immediate early promoter controlling the green fluorescent protein gene using the AdMax system (Microbix) (28). The adML-4 expressing IL-2 was previously described (29).

### Preparing Reovirus- and Adenovirus- Infected Cell Lysates or Media

L929 mouse fibroblasts (ATCC) were cultured in MEM (Sigma, M4655), 10% FBS (Sigma), 1 × non-essential amino acids (NEAA, Sigma, M7145), 1 mM sodium pyruvate solution (NaPyr, Sigma, S8636), and 1 × antibiotic antimitotic solution (AA, Sigma, A5955) at 37 °C with CO_2,_ until they reach 100% density in 4 plates of 150 cm^2^ surface area. For large- and medium-scale purifications, cells were transferred into 500 ml of JMEM (Sigma, M0518) with 2.2 g/L NaHCO_3_, 1.2 g/L HEPES, and 1 g/L glucose, in addition to FBS, NEAA, NaPyr, and AA as above. Cells were incubated in a spinner flask, spinning slowly 37 °C but without CO_2_. When cells reached 1 × 10^6^ cells/ml, they were diluted and propagated into more flasks as needed. Cells were infected at density of 1 × 10^6^ cells/ml by addition of reovirus at a multiplicity of infection (MOI) of 1. Cells were incubated, spinning at 37 °C, until they reached 70% cell death by trypan blue staining (~2–3 days). Cells were then collected by centrifugation at 1500 × g for 15 minutes to produce infected cell lysates. For small-scale purifications, L929 cells were seeded in 6 well plates (approximately 2 × 10^6^ cells/10 cm^2^ well equivalence), infected with reovirus at MOI of 3, incubated at 37 °C with CO_2_ for 20 hours, scraped into media, and concentrated by centrifugation at 1500 × g for 15 minutes. To generate adenovirus-infected lysates, HEK293 cells were infected with adenovirus and cultured until cytopathic effect exceeded 90%. Remaining cells were scraped into media and subjected to centrifugation to pellet cells and debris. Pellets were resuspended in adenovirus dilution buffer (10 mM Tris pH 8). A 10 cm^2^ confluent monolayer was used per condition. Lysates were frozen at −20 °C until subjected to purification by CsCl gradient ultracentrifugation or Capto Core 700 chromatography or in-slurry methods as described below.

### Coomassie Blue Staining, Silver Staining, and Western Blot analysis

Imperial™ Protein Stain (ThermoFisher scientific, 24615) was used for Coomassie Blue Staining. Silver staining was completed using the Silver Staining Plus^TM^ kit (Bio-Rad) according to manufacturer instructions. For western blot analysis, Reovirus proteins were detected using polyclonal rabbit anti-reovirus antibodies (generously provided by Dr. Patrick Lee, Dalhousie University, 1:10000) followed by Cy2-conjugated anti-rabbit secondary antibodies (Jackson Immunoresearch 111-545-144, 1:5000). The sample blot was re-probed with mouse anti-β-actin antibodies (Santa Cruz sc74471, 1:1000) and Cy5-conjugated anti-mouse secondary antibodies (Jackson Immunoresearch 115-175-166, 1:3000). A separate blot was incubated with rabbit anti-STAT1 (Cell Signalling 9167, 1:1000) followed by Cy5-conjugated anti-rabbit secondary antibodies. Protein bands were visualized at respective wavelengths using an ImageQuant LAS 4010 imager (GE Healthcare).

### Electron Microscopy

For negative staining, virus preparations were applied to carbon-coated formvar films and stained with 0.1% uranyl acetate. Micrographs were taken with a JEOL JEM-1230 transmission electron microscope.

## Additional Information

**How to cite this article**: James, K. T. *et al*. Novel High-throughput Approach for Purification of Infectious Virions. *Sci. Rep*. **6**, 36826; doi: 10.1038/srep36826 (2016).

**Publisher’s note:** Springer Nature remains neutral with regard to jurisdictional claims in published maps and institutional affiliations.

## Supplementary Material

Supplementary Information

## Figures and Tables

**Table 1:** Reovirus Purification Methods that Retain Virus Infectivity.

Method	Removal of Cellular Contaminants	Removal of Empty Virions	Approximate Time to Completion	Storage^1^	High Throughput Compatible	Scalable Purification^2^	Special Equipment Required
Crude Infected Cell Lysate	−	−	1 hr	Aliquots −20 °C	+++	NA	None
Media Harvest	+	−	1 hr	Aliquots −20 °C	+++	NA	None
100×Kg Spin +/− Sucrose Cushion	+	−	3 hr	Aliquots −20 °C	+	−	Ultracentrifuge
Density Gradient	++++	++++	3 days	4 °C	−	+	Ultracentrifuge
Capto Core 700 Chroma-tography	++++	−	5 hr	4 °C	−	+++	Chromatography System
Capto Core 700 In-slurry	+++	−	3 hr	4 °C	+++	++	None

^1^Aliquots and freezing at −20 °C is beneficial in all cases to reduce microbial contamination and for long term storage, respectively.

^2^Scalable reflects to the ability to efficiently purify virus from very small to very large sample volumes. NA, not applicable due to absence of purification.

**Table 2:** Plaque Titers of Unpurified and Purified Adenovirus.

Purification Method	Sample Storage Temp.	Virus Preparation Titers (Pfu/ml)^1^		
		Ad*dl309*	Ad*dl70.3*	Ad*GFP*
Unpurified Lysate	4 °C	9.78E + 08	1.83E + 08	3.15E + 08
	−80 °C	1.13E + 09	1.94E + 08	1.61E + 08
1	4 °C	1.79E + 09	1.88E + 08	2.78E + 08
	−80 °C	1.73E + 09	1.17E + 08	1.08E + 08
2	4 °C	2.41E + 09	nd	nd
	−80 °C	1.24E + 09	nd	nd
3	4 °C	1.97E + 09	2.36E + 08	5.21E + 08
	−80 °C	1.95E + 09	2.42E + 08	3.63E + 08
4	4 °C	1.24E + 09	nd	nd
	−80 °C	4.35E + 09	nd	nd

^1^nd, not determined.
