# Supplementary material for: Novel High-throughput Approach for Purification of Infectious Virions
Source: Sci Rep. 2016 Nov 9;6:36826. doi: 10.1038/srep36826 (PMC5101806; doi:10.1038/srep36826)
Supplement: Supplementary Information [file srep36826-s1.pdf]

Novel High-throughput Approach for Purification of Infectious Virions

Kevin T. James[1], Brad Cooney[2], Kate Agopsowicz[3#], Mary Ann Trevors[4#], Adil Mohamed[1], Don Stoltz[4], Mary Hitt[3], Maya Shmulevitz[1*],

[1]Department of Medical Microbiology and Immunology, Li Ka Shing Institute of Virology, University of Alberta, Edmonton, Alberta, Canada.

[2]GE Healthcare Canada, brad.cooney@ge.com.

[3]Department of Oncology, University of Alberta, Edmonton, Alberta, Canada

[4]Department of Microbiology and Immunology, Dalhousie University, Halifax, Nova Scotia, Canada

[#]Authors contributed equally to this work

Running Head: High-throughput Virus Purification

*Address correspondence to Maya Shmulevitz, shmulevi@ualberta.ca.

Supplementary Materials and Methods:

Virus purification by CsCl, Reovirus purification on Capto Core 700 chromatography columns, and Capto Core 700 slurry

(A) Purification by CsCl Gradient Ultracentrifugation

- *This protocol is according to 1L cultures. For 500ml cultures, use ½ vol.*
- *Proceed on ice.*
- *During sonication, do not let suspension froth.*
- *Store virus at 4°C, or aliquot and store at -20°C for long-term usage. Avoid freeze-thaw cycles.*

1. Resuspend cell pellet in 18ml of HO Buffer.
2. (Optional) Add Sodium Deoxycholate (10% solution in water) to a final concentration of 0.2%.
3. Vortex 5 minutes.
4. Incubate on ice for 20 minutes.
5. Add 18ml of Vertrel™ XF.
6. Sonicate on amplitude 70% for two pulses of 5 seconds each.
7. Subject sample to centrifugation at 4000 RPM for 10 minutes.
8. Transfer the top aqueous phase to a new tube.
9. Repeat steps 5-8.
10. Layer aqueous phase onto CsCl Gradient as follows:
    - 1/4th final volume of CsCl 1.4 g/cc
    - 1/4th final volume of CsCl 1.2 g/cc
    - 1/2 final volume of CsCl of aqueous phase from extraction above
11. Subject sample to high speed centrifugation at 112,500 x g for 5-18 hours.
12. To collect virus band, either (1) puncture tube with a 5 ml syringe and a needle below the band of interest, and slowly draw the virus into the syringe, or (2) slowly remove liquid using a p1000 pipettor from the top of the tube until the band of interest is reached, and collect.
13. Load virus into dialysis tubing (10 kD MWCO).
14. Dialyze against 3 sets of virus dilution buffer (VDB), first for >1 hour, then >3 hours, then >6 hours.
15. Collect virus from dialysis membrane.

□

(B) Purification by Capto700 Chromatography

- *This protocol is according to 1L cultures. For 500ml cultures, use ½ vol.*
- *Proceed on ice.*
- *During sonication, do not let suspension froth.*
- *Store virus at 4°C, or aliquot and store at -20°C for long-term usage. Avoid freeze-thaw cycles.*

1. Resuspend infected cell pellet in 6.5ml of Virus Dilution Buffer.
2. Vortex 2 minutes.
3. Let lysate sit on ice for 2 minutes.
4. Add 6.5ml of Vertrel™ XF.
5. Vortex 2 minutes.
6. Sonicate on amplitude 70% for two pulses of 5 seconds each.
7. Subject sample to centrifugation at 4000 RPM for 10 minutes.
8. Transfer the top aqueous phase to a new tube.
9. Filter through 0.45µm GD/x filter.
10. Pass sample through a Capto700 Column (e.g. AKTA Start chromatography system or a comparable Chromatography system) as follows:
    a) Attach Capto Core 700 1ml column via "drop to drop" connection to avoid introduction of air on to the column.
    b) Flush column at ~1ml/min with ddH_20 until stable UV and Conductivity.
    c) Add sample via Sample Loop or add larger samples via Sample Inlet.
    d) Add the A buffer line into Virus Dilution Buffer.
    e) Equilibrate the column using Virus Dilution Buffer until Conductivity and UV are stable.
    f) Set fraction collector to collect 0.5ml fractions.
    g) Load sample onto the column.
    h) Virus should appear in the first 5-10 fractionation tubes, as indicated by a rise in UV and should be validated by other methods (e.g. western blot analysis, plaque titration, etc.).
    i) Clean the column to permit reuse with 3-5 column volumes of 30% Isopropanol and 1.0M NaOH.
11. (Optional) Concentrate virus using VivaSpin (100 kD MWCO) sample concentrators.

(C) Purification Capto700 Slurry

- *This protocol is per 6 x 10^6 cells infected with reovirus as in (A) or 1 x 10^7infected cells with adenovirus.*
- *This protocol is scalable to larger or smaller cell lysates. Adjust volumes according to relative number of cells.*
- *Proceed on ice.*
- *Do NOT add protease inhibitors, as they can sometimes interfere with reovirus infectivity.*
- *To prepare 50% Capto700 Slurry, spin Capto700 resin at 800 x g for 5 minutes and wash 2 times with equal-to-resin volume of Virus Dilution Buffer. Store as 50% slurry in VDB.*
- *Store virus at 4°C, or aliquot and store at -20°C for long-term usage. Avoid freeze-thaw cycles.*

1. Resuspend cell pellet in 350µl of Virus Dilution Buffer.
2. Freeze-thaw 2 times.
3. (Optional) Add 350µl of Vertrel™ XF, vortex for 2 minutes, spin at 1000 x g for 10 minutes, and transfer top aqueous phase. If choosing this option, proceed to step 12.
4. Remove nuclei and debris by centrifugation at 800 x g for 10 minutes.

5. Add 50-100µl of 50% Capto700 Slurry.
6. Mix sample end-over-end for 45 minutes at room temperature.
7. Subject sample to centrifugation at 800 x g for 10 minutes.
8. Transfer the top phase to a new tube.
9. Repeat steps 5-8, 1-2 times for increasing purity
10. (Optional) To clear all Capto700 resin, pass sample through an Illustra MicroSpin column at 800 x g for 5 minutes.

RECIPES

1. JMEM L929 Suspension Culture Media

**REAGENT**	**Per L**
JMEM (Sigma-Aldrich, M0518) Powder	11g
Sodium bicarbonate ($NaHCO_3$)	2.2g
HEPES (4-(2-hydroxyethyl)-1-piperazineethanesulfonic acid)	1.2g (or 1.3g if Sodium salt)
Glucose (or dextrose)	1g
ddH_2O	900ml

Mix Components. Adjust to pH 7.2 with 10N NaOH (~375µl of 10N NaOH per L). Add dd$H_2$0 to 1 L with water. Filter sterilize and store at 4°C.

2. Supplemented JMEM ($JMEM^{+++}$)

**REAGENT**	**Per 500ml**
FBS	25ml (5% final)
Non-Essential Amino Acids Solution 100X (NEAA)	5ml
Sodium Pyruvate	5ml
Antibiotic/Antimycotic	5ml

Supplement JMEM when ready for use.

3. HO Buffer

**REAGENT**	**Per 100ml**	**[Final]**
1M Tris pH 7.4	1ml	10mM

5M NaCl	5ml	250mM
β-mercaptoethanol	67µl	
ddH_2O	To 100ml	

Filter sterilize through standard 0.45µm syringe filter.

4. Virus Dilution Buffer (VDB) for reovirus

**REAGENT**	**Per 1 L**	**[Final]**
5M NaCl	30ml	150mM
1M $MgCl_2$	15ml	15mM
1M Tris pH 7.4	10ml	10mM
ddH2O	To 1L	

Filter sterilize through standard 0.45µm bottle filter.

5. Virus Dilution Buffer (VDB) for adenovirus

REAGENT	Per 1 L	[Final]
1M Tris pH 8.0	10ml	10mM
ddH2O	To 1L	

6. CsCl Solutions

For 1.2g/cc, mix 33.3g CsCl with 100ml of 10mM Tris pH7.4

For 1.4g/cc, mix 67g CsCl with 100ml of 10mM Tris pH7.4

Stir solutions and allow them to reach room temperature. Using a calibrated scale, bring solutions to correct density by adding either CsCl or 10mM Tris pH7.4. Filter sterilize through standard 0.45µm bottle filter.

ADDITIONAL REAGENTS and EQUIPMENT

**REAGENTS and EQUIPMENT**	**Catalogue Number**	**Source**
Vertrel™ XF fluorinated solvent	Vertrel™ XF	Dymar Chemicals Ltd., Mississauga, ON, Canada

AKTA Start	29-0220-94 + 29-0230-51	GE Healthcare
Capto Core 700 1ml column	17-5481-51 (5 pieces)	GE Healthcare
0.45µm Whatman filter (regenerated Cellulose GD/X filters)	6882-2504	GE Healthcare
Capto Core 700 slurry (25ml)	17-5481-01	GE Healthcare
Illustra MicroSpin columns	27-3565-01 (50 pieces)	GE Healthcare
(Optional) VivaSpin 6 (100,000 MWCO) 2-6mls sample concentrator	28-9323-19 (25 pieces)	GE Healthcare
(Optional) VivaSpin 2 (100,000 MWCO) 400µl – 2ml sample concentrator	28-9322-58 (25 pieces)	GE Healthcare

REFERENCES

1. Bett, A.J., Haddara, W., Prevec, L. & Graham, F.L. An efficient and flexible system for construction of adenovirus vectors with insertions or deletions in early regions 1 and 3. *Proc Natl Acad Sci U S A* **91**, 8802-8806 (1994).
2. Ng, P., Parks, R.J., Cummings, D.T., Evelegh, C.M. & Graham, F.L. An enhanced system for construction of adenoviral vectors by the two-plasmid rescue method. *Hum Gene Ther* **11**, 693-699 (2000).
3. Chaurasiya, O.S., Kumar, L. & Sethi, R.S. An infant with milky blood : an unusual but treatable case of familial hyperlipidemia. *Indian J Clin Biochem* **28**, 206-209 (2013).
